# A call to encourage curricular research publications by medical students

**DOI:** 10.5116/ijme.5842.fc6e

**Published:** 2016-12-18

**Authors:** Ahmed Abu-Zaid, Israa Bamogaddam, Lubna AlBader, Lama AlFakhri, Akram Nurhussen

**Affiliations:** 1 College of Medicine, Alfaisal University, Riyadh, Saudi Arabia

**Keywords:** Curricular research, publications, medical students

There is a rapidly mounting propensity towards incorporating structured scientific research training into undergraduate medical education.^1^ As a result, undergraduate medical students participate in various curricular faculty-mentored and student-run scholarly research projects. Some students produce, to a greater degree, technically correct and scientifically sound pieces of literature that can be potentially transformed into valuable publications. However, not many students have the chance to disseminate their curricular research activities through publications to the scientific community. Two legitimate questions pop up in mind: 1) what is the reason behind this production-outcome mismatch, and 2) who should be held responsible?

Student research activities are useless if conducted without knowledge dissemination, favorably through publication in professional peer-reviewed journals. Student research publications should not be viewed as “inferior” scientific evidence as they can provide distinguished intellectual perspectives with a significant contribution to the ever-expanding body of science. This belief is supported by the welcoming of all professional scientific journals to accept research contributions from all researchers, including medical students.

Medical students should move away from perceiving curricular research activities as mere occasions to simply accomplish tasks and satisfy the grade-related requirements of a particular course or an assignment. Instead, students should aim towards meaningful research outcomes, both qualitatively (learning) and quantitatively (publication). Motivation plays a pivotal role in the process, especially when the student’s motivation is intrinsic (that is, there is an interest in the subject of research and a desire to disseminate findings) rather than extrinsic (that is, there is an interest in only meeting good evaluation requirements). In fact, owning one's research question or any question is apt to lead to true learning and publication. In brief, medical students should develop positive scholarly attitudes to view these curricular research activities as golden opportunities to envision life as quasi-scholars and endeavor to publish their research findings. 

Course instructors play central roles in the process, too. They should provide continuous feedback and thorough step-by-step guidance on how students’ research classwork can be translated into layouts suitable for publication in professional scientific journals. Most importantly, the instructors’ feedback to students should be mostly developmental for the sake of learning and maximizing the likelihood of publication (pedagogical) rather than solely judgmental and condemning students to course grades (evaluative). The medical curriculum should substantially reinforce the roles addressed above of course instructors.

Publishing manuscripts during the early years of medical education is correlated with more engagement in future research experiences.^2^ These future research experiences allow students to advance their research competencies, further deepen their interest in research, and robustly encourage them to consider the largely neglected clinician-investigator and academic medicine professions.^2^ Even if the medical student does not wish to pursue a research activity/career, one should be well-prepared to read scientific journals critically, as well as to decide on proper clinical decisions based on critical interpretation of scientific evidence. Interesting future research directions include 1) exploring whether research publications should be mandatory core competencies of the medical curriculum, 2) evaluating the outcome of students’ curricular versus extracurricular research publications, and 3) whether students who specifically publish manuscripts are more likely to become future physician-scientists.
